# Metabolomics reveals arbuscular mycorrhizal fungi-mediated tolerance of walnut to soil drought

**DOI:** 10.1186/s12870-023-04111-3

**Published:** 2023-02-28

**Authors:** Ying-Ning Zou, Qiu-Yun Qin, Wen-Ya Ma, Li-Jun Zhou, Qiang-Sheng Wu, Yong-Jie Xu, Kamil Kuča, Abeer Hashem, Al-Bandari Fahad Al-Arjani, Khalid F. Almutairi, Elsayed Fathi Abd-Allah

**Affiliations:** 1 Tibet Plateau Walnut Industry Research Institute / College of Horticulture and Gardening, Yangtze University, Jingzhou, Hubei 434025 China; 2 Faculty of Science, Department of Chemistry, University of Hradec Kralove, Hradec Kralove, 50003 Czech Republic; 3 Hubei Academy of Forestry, Wuhan, 430075 China; 4 Botany and Microbiology Department, College of Science, King Saud University, P.O. Box 2460, Riyadh, 11451 Saudi Arabia; 5 Plant Production Department, College of Food and Agricultural Sciences, King Saud University, P.O. Box 2460, Riyadh, 11451 Saudi Arabia

**Keywords:** Juglone, Metabolite, Nut fruits, Phenylalanine, Walnut, Water deficit, Symbiosis

## Abstract

**Background:**

Arbuscular mycorrhizal fungi (AMF) have a positive effect on drought tolerance of plants after establishing reciprocal resymbiosis with roots, while the underlying mechanism is not deciphered. Metabolomics can explain the mechanism of plant response to environmental stress by analyzing the changes of all small molecular weight metabolites. The purpose of this study was to use Ultra High Performance Liquid Chromatography Q Exactive Mass Spectrometer to analyze changes in root metabolites of walnut (*Juglans regia*) after inoculation with an arbuscular mycorrhizal fungus *Diversispora spurca* under well-watered (WW) and drought stress (DS).

**Results:**

Sixty days of soil drought significantly inhibited root mycorrhizal colonization rate, shoot and root biomass production, and leaf water potential in walnut, while AMF inoculation significantly increased biomass production and leaf water potential, accompanied by a higher increase magnitude under DS versus under WW. A total of 3278 metabolites were identified. Under WW, AMF inoculation up-regulated 172 metabolites and down-regulated 61 metabolites, along with no changes in 1104 metabolites. However, under DS, AMF inoculation up-regulated 49 metabolites and down-regulated 116 metabolites, coupled with no changes in 1172 metabolites. Among them, juglone (a quinone found in walnuts) as the first ranked differential metabolite was up-regulated by AMF under WW but not under DS; 2,3,5-trihydroxy-5–7-dimethoxyflavanone as the first ranked differential metabolite was increased by AMF under DS but not under WW. The KEGG annotation showed a large number of metabolic pathways triggered by AMF, accompanied by different metabolic pathways under WW and DS. Among them, oxidative phosphorylation and phenylalanine metabolism and biosynthesis were triggered by AMF in response to WW and DS, where N-acetyl-L-phenylalanine was induced by AMF to increase under DS, while decreasing under WW.

**Conclusion:**

This study provides new insights into the metabolic mechanisms of mycorrhiza-enhanced drought tolerance in walnuts.

## Background

Walnut (*Juglans regia* L.) is one of the most important nut crops rich in ω-3 fatty acids and has been grown in Asia, South America, North America and Europe [1]. Among them, China is the largest producer and consumer of walnuts. Among the commercial varieties of walnuts in China, Qingxiang is widely planted in Hubei, Xinjiang, Shandong, and Hubei provinces in China because of its strong growth potential, early maturity, good productivity, aroma, and thin nut shell [2]. However, Qingxiang is more sensitive to soil drought than other varieties, along with a high water demand [3], resulting in frequent exposure to soil drought stress (DS) [4, 5]. Therefore, it is particularly urgent to enhance the drought tolerance of Qingxiang walnut.

Soil drought is a major abiotic factor limiting crop growth and yield [6]. It is estimated that 50% of crop losses are the result of abiotic stress, of which 10% is attributed to soil drought [7]. Moreover, soil drought has been prolonged in some arid regions, and the severity of soil water deficit has continued to increase in recent years [7]. Therefore, there is an urgent need to find solutions to improve plant growth under soil water deficit.

Arbuscular mycorrhizal fungi (AMF), a beneficial group of fungi of the Glomeromycotina, inhabit in various soils and can colonize the roots of a large number of terrestrial plants including walnut to establish reciprocal symbionts [8]. A key feature of mycorrhizal symbiosis is that they help the host plant obtain nutrients and water from the soil [9]. In addition, earlier studies have also shown that AMF could enhance the drought tolerance of host plants, while the mechanisms involved have not been fully elucidated, although various mechanisms, such as direct water uptake by mycorrhizal extraradical hyphae, improvement of nutrient elements, activation of osmotic regulation and antioxidant defense system, as well as potential molecular mechanisms have been reported [10–12]. For example, AMF could trigger the expression of *catalase* and *peroxidase* genes in soybean plants under soil drought conditions [13]. Among them, metabolites such as sugars, fatty acids, polyamines, late embryogenesis abundant proteins, and hormones are involved in mycorrhizal improvement of plant stress tolerance and interact with each other [14–16]. In soybean, co-inoculation with AMF and *Bacillus amyloliquefaciens* also increased phenol, flavonoid, soluble sugars, lipids, protein, and oil contents under drought stress conditions [17]. Therefore, a comprehensive analysis of metabolites can reveal the metabolic pathways of mycorrhizal effects on plant drought tolerance.

Metabolomics is the analysis of changes in all small-molecule metabolites (molecular weight less than 1000), such as sugars, lipids, amino acids and nucleotides, in fresh tissue samples during a specific physiological period, thus providing global changes of metabolites of organisms after environmental stresses [18]. Metabolomics has been used to uncover the mechanisms by which mycorrhizae enhance plant tolerance to abiotic stress [19, 20]. Rivero et al. [21] used liquid chromatography and electrospray ionization mass spectrometry to analyze changes in metabolites of *Solanum lycopersicum* plants inoculated with *Funneliformis mosseae*, *Rhizoglomus irregulare* and *Claroideoglomus etunicatus* from different habitats and found that mycorrhizal symbionts enhanced the metabolic plasticity of plants against salt stress. Bernardo et al. [22] found in metabolomics that metabolites associated with oxidative stress were involved in hormone crosstalk as well as brassinosteroids biosynthesis pathways in *F*. *mosseae*-enhanced resistance to water deficit in *Triticum durum* plants. Hence, changes in metabolomics allow deciphering mycorrhizal function in adapting to abiotic stress.

Earlier studies showed that a certain number of arbuscular mycorrhizal fungal populations existed in the rhizosphere of walnut [23], and inoculation of AMF on walnut promoted nutrient acquisition, with *Diversispora spurca* being a dominant strain [24, 25]. The results of Behrooz et al. [26, 27] also indicated that AMF improved the adaptation of walnut plants to soil DS by increasing total phenols, proline, nutrient nutrients, etc. This indicated that AMF have the potential ability to enhance drought resistance of walnut, but the related mechanisms are not understood. The objective of this study was to perform non-targeted metabolomic analysis of walnut roots inoculated with and without AMF under well-watered (WW) and DS conditions to elucidate the metabolic mechanisms by which AMF help walnut tolerate drought.

## Results

### Changes in root AMF colonization and biomass production

**Fig. 1:**
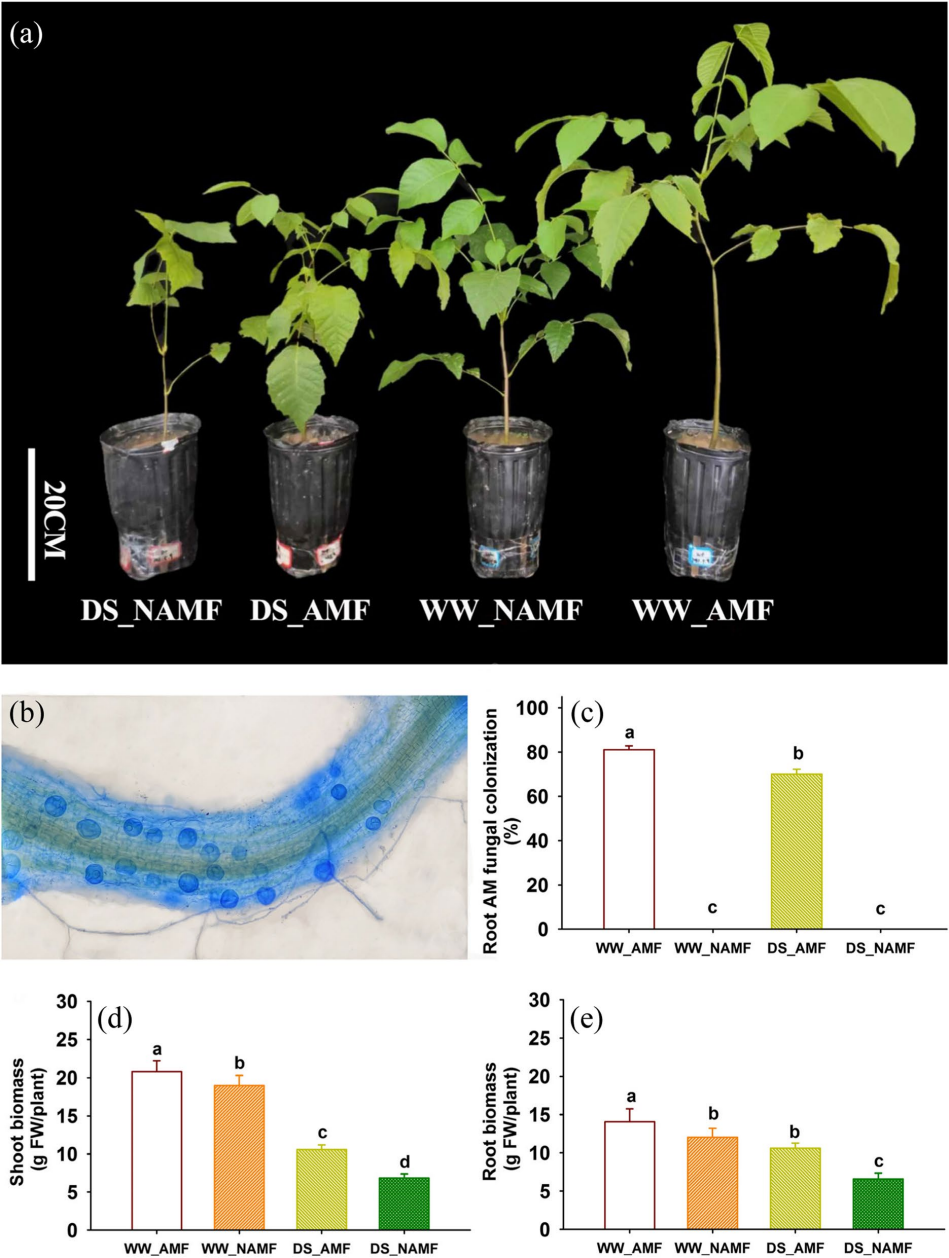
Changes in plant growth performance (**a**), root mycorrhizal colonization (**b**), root mycorrhizal colonization rate (**c**), and shoot (**d**) and root (**e**) biomass production of *Juglans regia* inoculated with *Diversispora spurca* under well-watered and drought stress conditions. Data (means ± SD, *n* = 4) followed by different letters above the bars indicate significant (*P* < 0.05) differences. Abbreviations: DS_AMF, the treatment with drought stress and* D*. *spurca* inoculation; DS_NAMF, the treatment with drought stress and non-inoculation of *D*. *spurca*; WW_AMF, the treatment with *D*. *spurca* inoculation and well-watered; WW_NAMF, the treatment with well-watered and non-inoculation of *D*. *spurca*

Soil drought inhibited shoot and root biomass production, independent of AMF inoculation (Fig. 1a, d, e). However, *D*. *spurca* inoculation improved shoot and root biomass production by 9.4% and 16.7% under WW and 54.8% and 60.7% under DS, respectively. Signs of *D*. *spurca* colonization were found in roots of inoculated walnut plants (Fig. 1b). There was no mycorrhizal colonization of the roots of the non-mycorrhizal plants. Root mycorrhizal colonization rate of *D*. *spurca*-inoculated walnut ranged from 70.01% to 81.00%, and soil drought treatment significantly inhibited root mycorrhizal colonization rate by 13.6% (Fig. 1c).

### Changes in leaf water potential

**Fig. 2:**
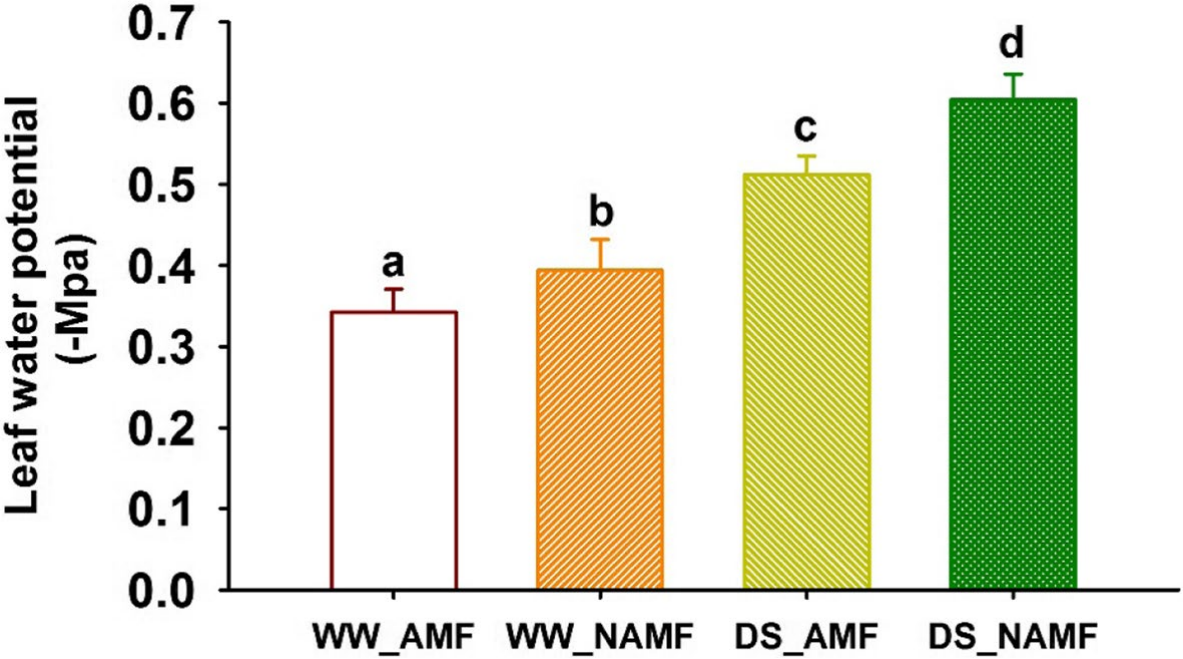
Changes in leaf water potential of *Juglans regia* inoculated with *Diversispora spurca* under well-watered and drought stress conditions. Data (means ± SD, *n* = 4) followed by different letters above the bars indicate significant (*P* < 0.05) differences. The abbreviations were shown in Fig. 1

Leaf water potential was significantly inhibited by 49.71% in AMF-inoculated plants and 53.30% in non-AMF-inoculated plants under soil drought versus WW treatment (Fig. 2). AMF colonization significantly increased leaf water potential by 13.20% under WW and 15.23% under DS, respectively.

### Principal component analysis (PCA)

**Fig. 3:**
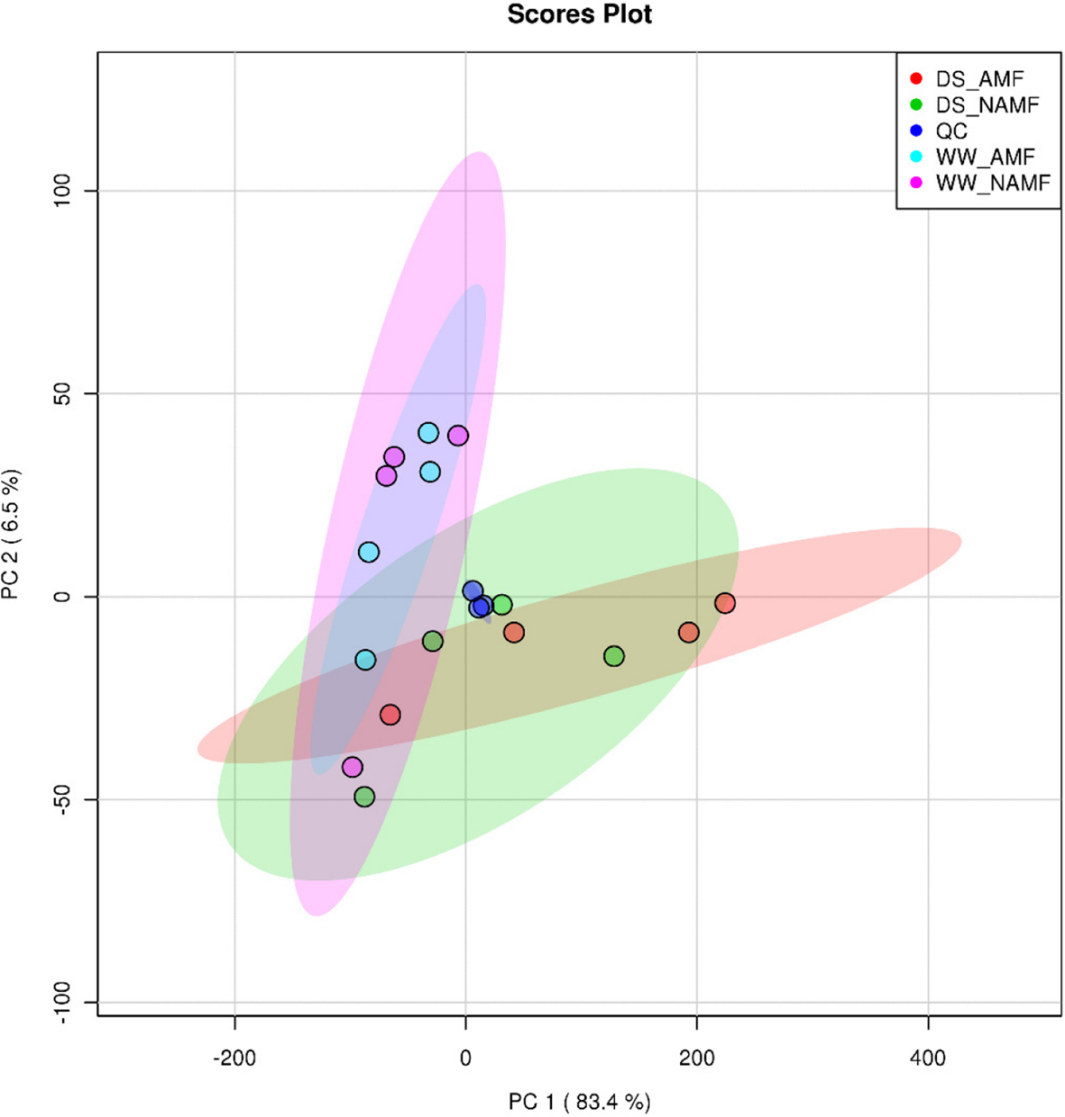
Principal component analysis of metabolites in roots of *Juglans regia*. Abbreviations: PC, principal component; QC, quality control. Other abbreviations were shown in Fig. 1

PCA was used to observe the separation trend between treatments in the experiment and whether there were outliers, and to reflect the degree of variation between and within treatments from the original data. The treatments in this study were divided into two principal components (PCs), of which PC1 in the X axial was 83.4% and PC2 in the Y axial was 6.5% (Fig. 3). PC1 mainly distinguished between WW and DS treatments.

### Orthogonal partial least squares discriminant analysis (OPLS-DA)

**Fig. 4:**
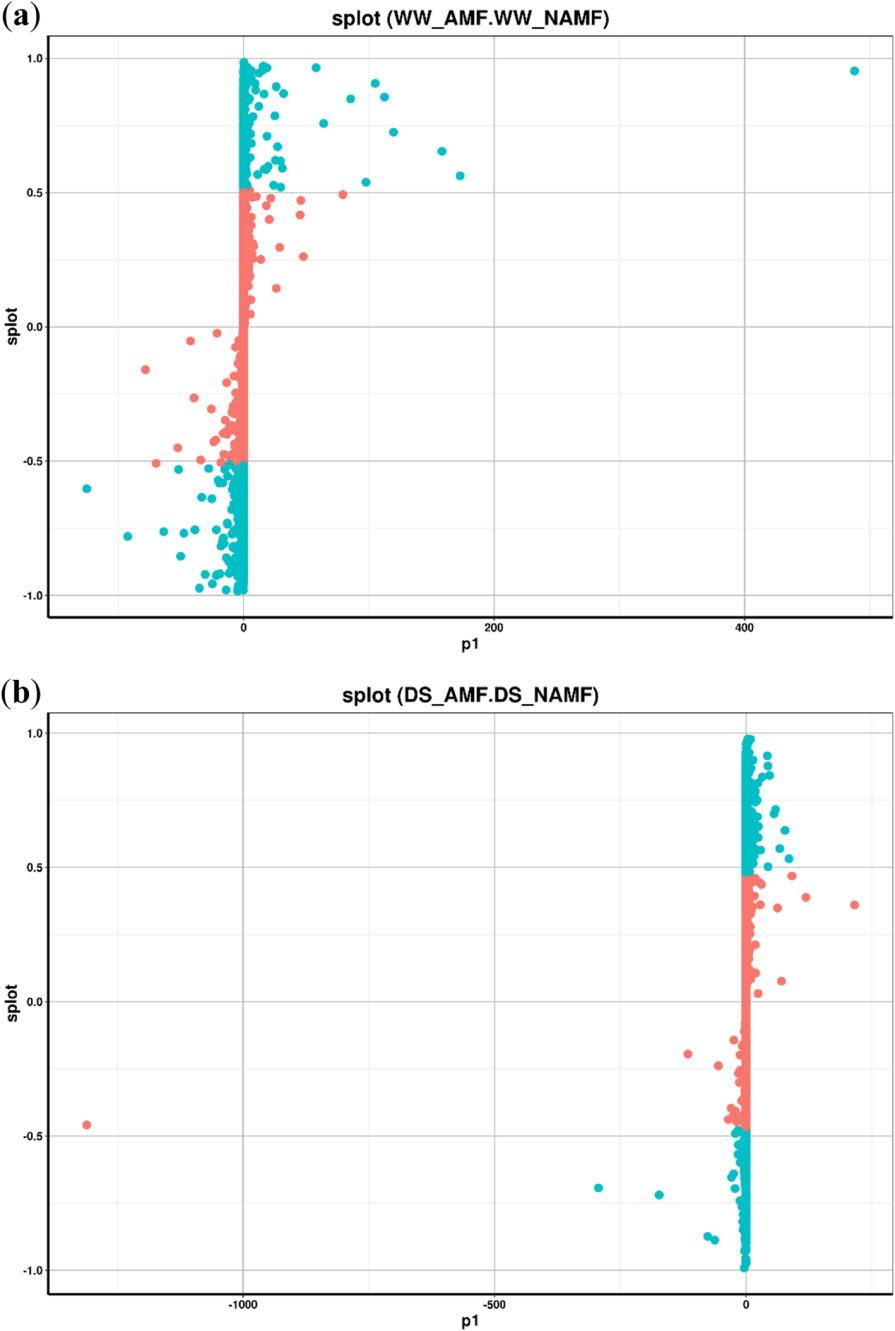
S-plot of orthogonal partial least squares discriminant analysis for metabolites between WW_AMF and WW_NAMF (**a**) and DS_AMF and DS_NAMF (**b**). Red dots and green dots indicate that these metabolites had a VIP value of < 1 and > 1, respectively. The abbreviations were shown in Fig. 1

To obtain a high level of treatment separation and get a better understanding of variables responsible for classification, OPLS-DA was applied. Here, T score (11.2%) denoted the predicted PC score of the PC1, and orthogonal T score (26%) showed the orthogonal PC score. Based on the model validation, R2 (the model explanation rate) and Q2 (the predictive ability of the model) were 0.998 and 0.732, along with *p* < 0.005, indicating that the measured data were reliable. In Fig. 4, green dots (VIP > 1) were selected as the differential metabolites, of which AMF inoculation under WW significantly triggered a large number of differential metabolites (Fig. 4a), while under DS, AMF inoculation produced a relatively small number of differential metabolites (Fig. 4b).

### Changes in root differential metabolites

**Fig. 5:**
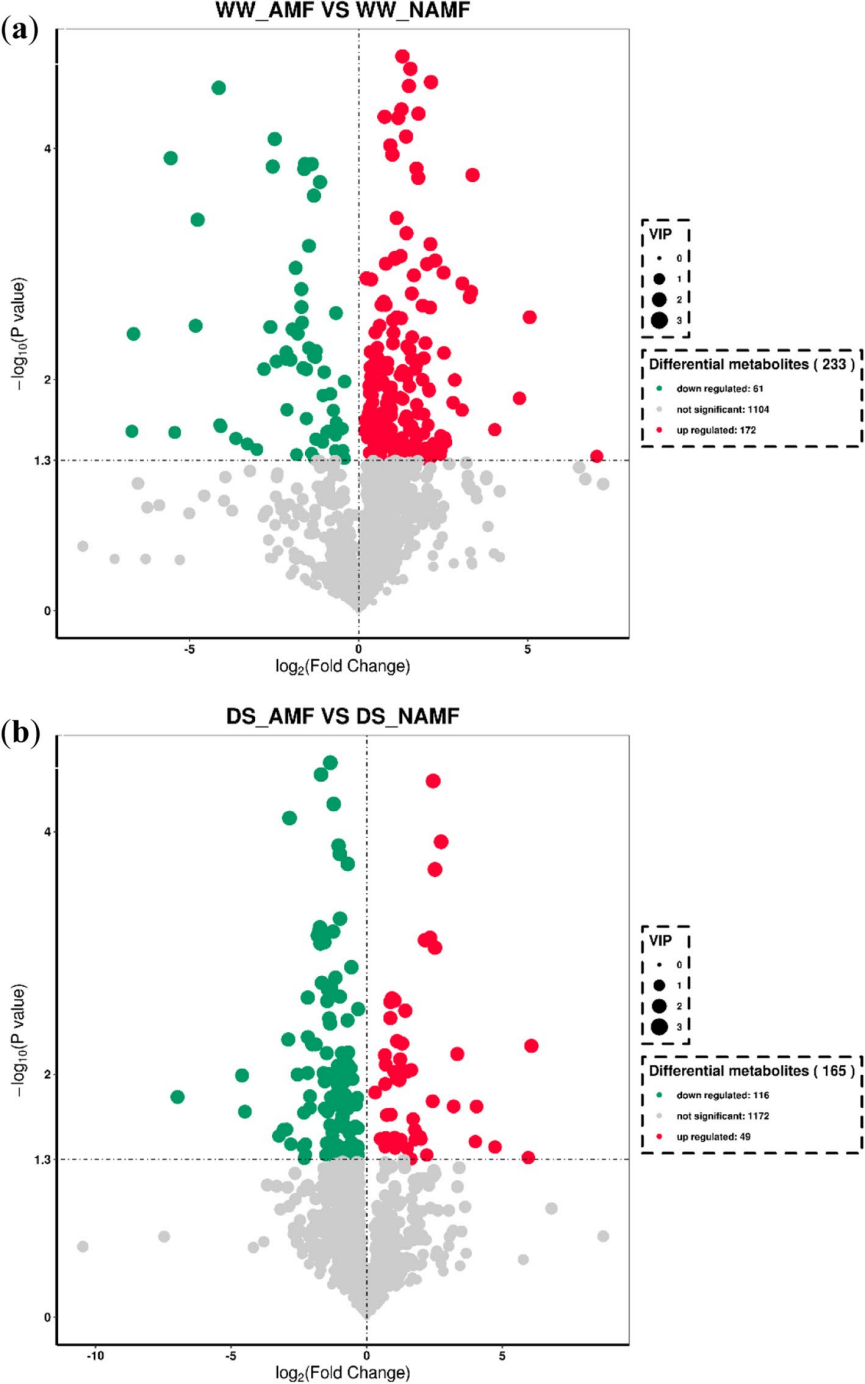
Number of differential metabolites in roots of *Juglans regia* between WW_AMF and WW_NAMF (**a**) and DS_AMF and DS_NAMF (**b**). The abbreviations were shown in Fig. 1

In this study, a total of 3278 metabolites were identified, among which 891 secondary metabolites were precisely detected. Under WW, a total of 1337 secondary metabolites were identified, along with 233 differential metabolites (61 metabolites down-regulated and 172 metabolites up-regulated) and 1104 unchanged metabolites (Fig. 5a). Similarly, under DS, 1337 metabolites were further identified, of which 116 metabolites were down-regulated and 49 were up-regulated, accompanied by no significant change in 1172 metabolites (Fig. 5b). This indicated that the differential metabolites triggered by AMF under DS (165) were significantly lower than under WW (233), and the number of up-regulated metabolites was dramatically increased from 49 under DS to 172 under WW.

**Fig. 6:**
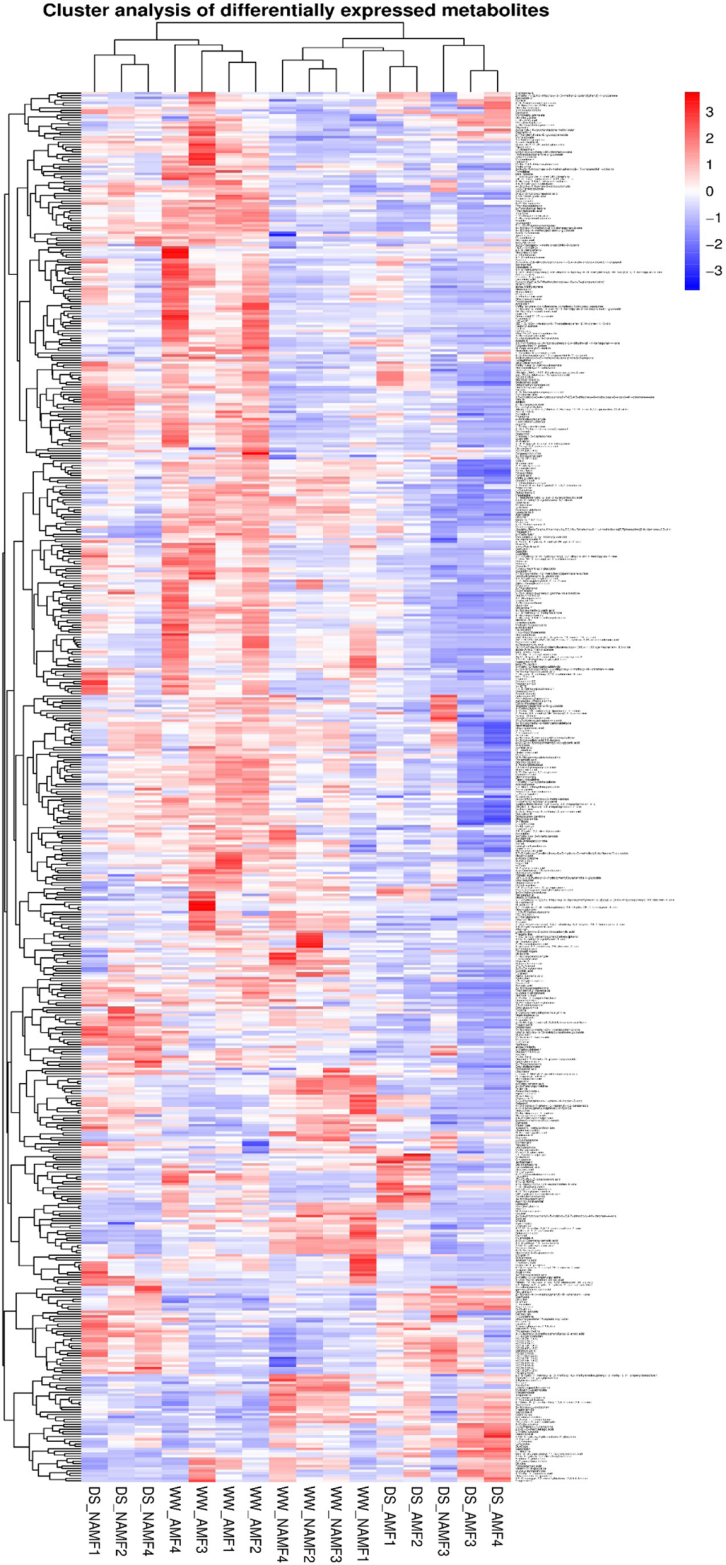
Hierarchical cluster analysis in roots of *Juglans regia* inoculated with *Diversispora spurca* under well-watered and drought stress conditions. The abbreviations were shown in Fig. 1

Differential metabolites often have biological similarity or complementarity in results and functions, and are positively/negatively regulated by the same metabolic pathway. Hierarchical clustering analysis of differential metabolites is helpful to group the metabolites with the same characteristics and finds the characteristics of metabolites variation between treatments. Figure 6 clearly showed the differences and hierarchical clustering results of these associated differential metabolites among treatments.

**Fig. 7:**
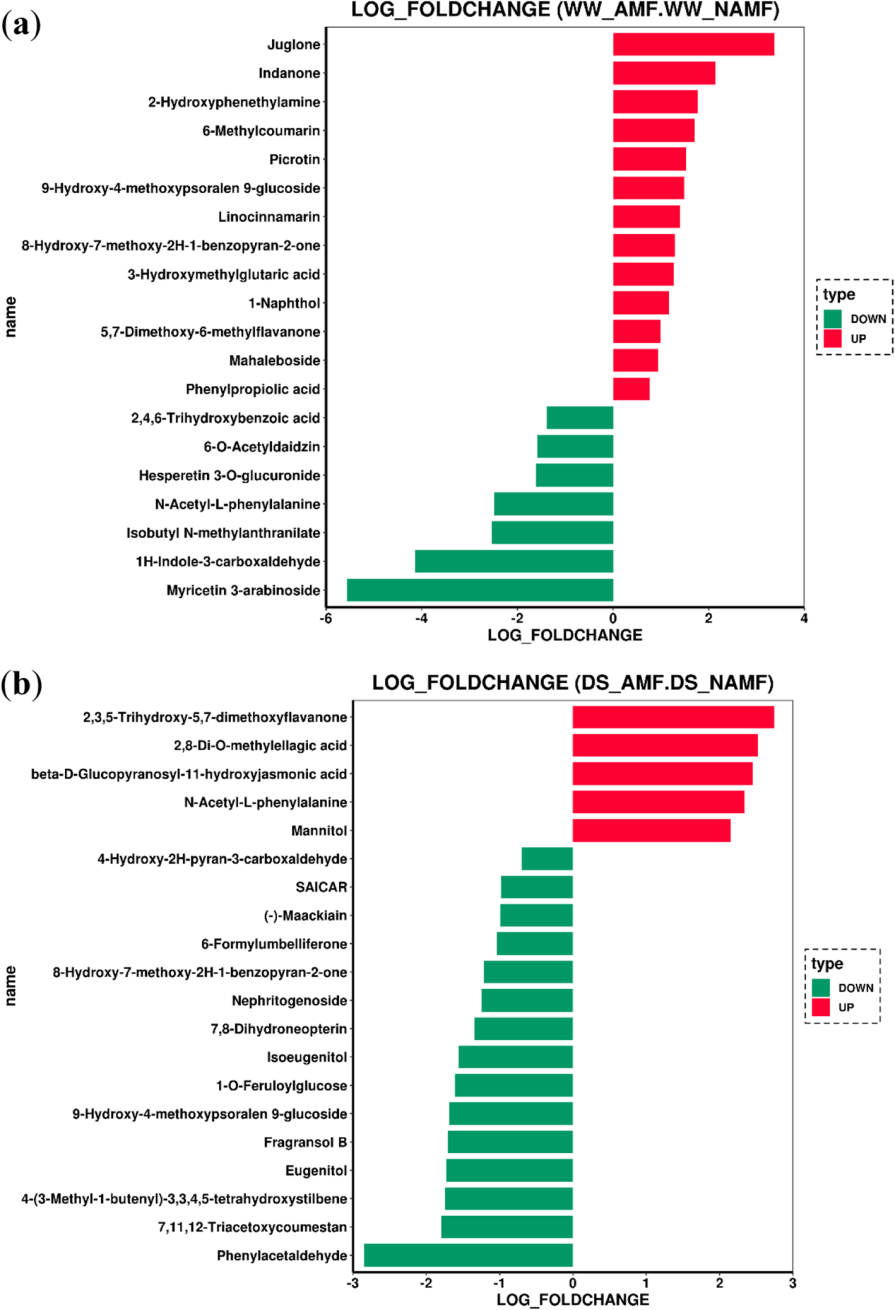
The top 20 differential metabolites in roots of *Juglans regia* between WW_AMF and WW_NAMF (**a**) and DS_AMF and DS_NAMF (**b**). The abbreviations were shown in Fig. 1

The results for the top 20 differential metabolites in terms of fold change showed that under WW, the up-regulated differential metabolites were arranged as the trend of juglone > indanone > 2-hydroxyphenethylamine > 6-methylcoumarin > picrotin > 9-hydroxy-4-methoxypsoralen-9-glucoside > linocinnamarin > 8-hydroxy-7-methoxy-2H-benzopyran-2-one > 3-hydroxymethylglutaric acid > 1-naphthol > 5,7-dimethoxy-6-methylflavanone > mehaleboside > phenylpropiolic acid in the descending order; the down-regulated differential metabolites were listed as the trend of myricetin 3-arabinoside > 1H-indole-3-carboxaldehyde > isobutyl N-methylanthranilate > N-acetyl-L-phenylalanine > 3-O- glucuronide hesperetin > 6-O-acetyldaidzin > 2,4,6-trihydroxybenzoic acid in the descending order (Fig. 7a).

On the other hand, the results of the top 20 differential metabolites triggered by AMF under DS conditions in terms of fold change indicated that the up-regulated differential metabolites were ranked as the order of 2,3,5-trihydroxy-5–7-dimethoxyflavanone > 2,8-di-O-methylellagic acid > β-D glucopyranosyl-11-hydroxyjasmonic acid > N-acetyl-L-phenylalanine > mannitol in the decreasing order; the down-regulated differential metabolites were ranked as the trend of phenylacetaldehyde > 7,11,12- triacetoxycoumestan > 4-(3-methyl-1-butenyl)-3,3,4,5-tetrahydroxystilbene > eugenitol > fragansol B > 9-hydroxy-4-methoxypsoralen 9-glucoside > 1-O-feruloylglucose > isoeugenitol > nephritogenoside > 8-hydroxy-7-methoxy-2H-1-benzopyran-2-one > 6-formylumbelliferone > (-)-maackiain > saicar > 4-hydroxy-2H-pyran-3-carboxaldehyde with a decreasing trend (Fig. 7b).

### Metabolic pathway analysis (KEGG) of differential metabolites

**Fig. 8:**
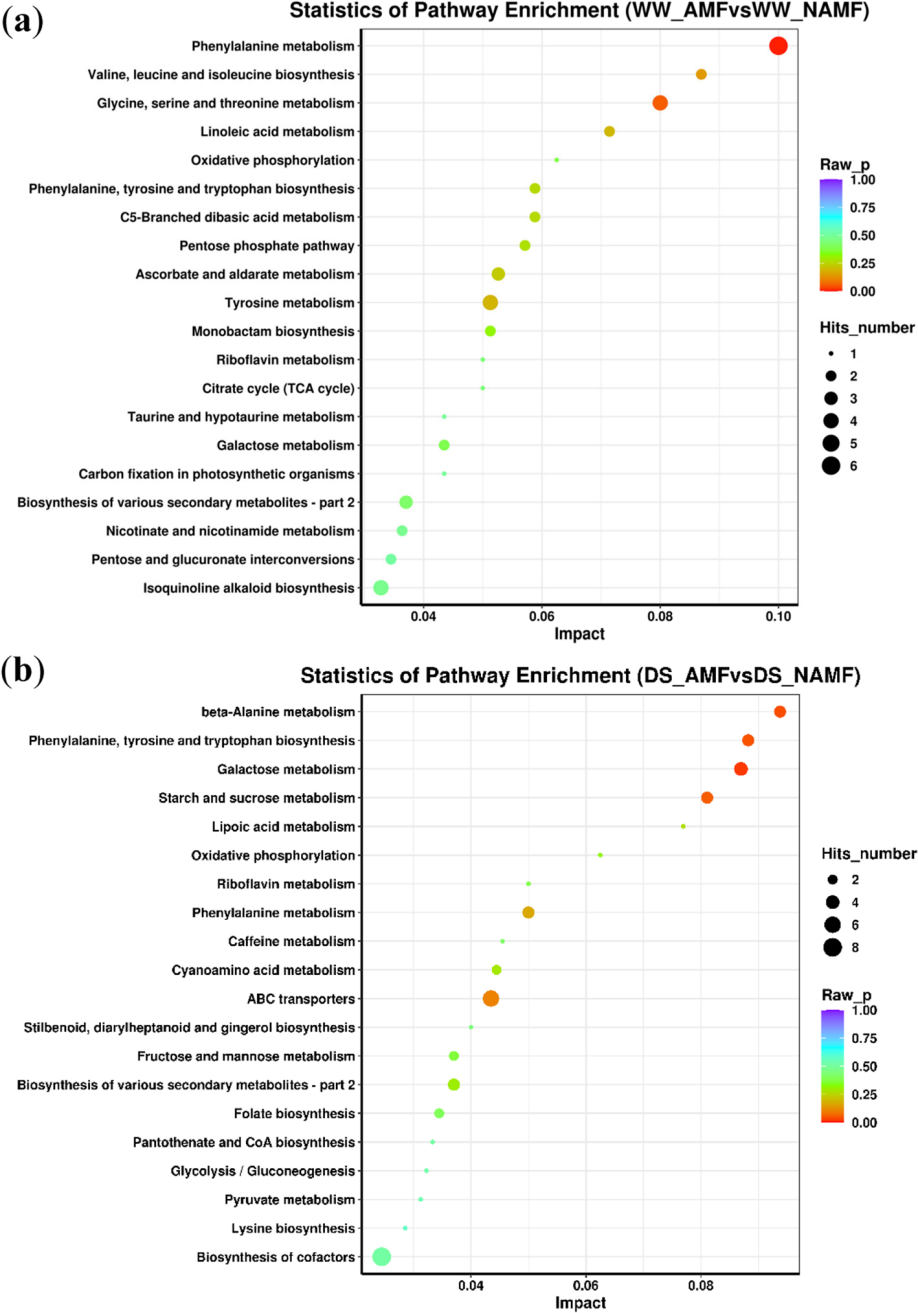
The top 20 metabolic pathways of differential metabolites annotated by KEEG in roots of *Juglans regia* between WW_AMF and WW_NAMF (**a**) and DS_AMF and DS_NAMF (**b**). The abbreviations were shown in Fig. 1

The differential metabolites were annotated using the KEGG database.The KEGG annotation showed that the top six differential metabolic pathways triggered by AMF under WW were phenylalanine metabolism, valine, leucine and isoleucine biosynthesis, glycine, serine and threonine metabolism, linoleic acid metabolism, oxidative phosphorylation, and phenyllanine, tryosine and tryptophan biosynthesis (Fig. 8a). Under DS, the top six differential metabolic pathways triggered by AMF were β-alanine metabolism, phenylalanine, tryosine and trytophan biosynthesis, galactose metabolism, starch and sucrose metabolism, lopoic acid metabolism, and oxidative phosphorylation (Fig. 8b).

## Discussion

The present study showed that the soil drought treatment significantly inhibited the biomass of walnut and root mycorrhizal colonization rate, while AMF still promoted the shoot and root biomass production and leaf water potential, and the promoting effect under DS was more prominent than under WW. This is consistent with the results of Liang et al. [19] in trifoliate orange inoculated with *Rhizophagus intraradices* under soil drought. This is explained by the fact that the absorption rate of water by AMF under drought was much higher than that under sufficient water [28]. Therefore, it is necessary to strengthen the management of indigenous AMF in walnut orchard located in mountainous areas for increased AMF diversity and root AMF colonization rate.

The present study showed that AMF inoculation triggered 233 differential metabolites (172 up-regulated metabolites and 61 down-regulated metabolites) under WW, whereas AMF inoculation induced 165 differential metabolites (49 up-regulated metabolites and 116 down-regulated metabolites) under DS. This suggests that AMF distinctly altered walnut metabolites, with a decrease in the number of differential metabolites under DS, accompanied by a significant decrease in the number of up-regulated metabolites and a significant increase in the number of down-regulated metabolites in response to soil drought. Liang et al. [19] also found that mycorrhiza-regulated differential metabolites decreased in trifoliate orange under drought than under WW, but the number of up-regulated metabolites increased and the number of down-regulated metabolites decreased. Yang et al. [20] found 44 up-regulated metabolites and 18 down-regulated metabolites in *Puccinellia tenuiflora* plants by *R*. *intraradices* under saline conditions. These results suggest that AMF altered walnut  secondary metabolism in response to drought, which is different from drought-sensitive trifoliate orange and halophyte *Puccinellia tenuiflora* in response to abiotic stress. Therefore, it is concluded that mycorrhizal alteration of plant metabolism depends on the host species as well as the AMF species.

Many trees in the Juglandaceae family produce a juglone, which is naturally inhibitory to some intercropping species (e.g. maize) in walnut orchards [29]. The present study revealed that under WW, but not under DS, AMF significantly (*P* < 0.05) increased root juglone levels of walnut by 3.37-fold (log2 fold-change; the same below), which was the highest up-regulated metabolite under WW. Mortier et al. [25] also found that mycelium of mycorrhizal symbiosis could transfer the juglone of walnut to the rhizosphere, which in turn inhibited the growth of the adjacent plant tomato plants. Thus, under sufficient water supply conditions, AMF can contribute to the allelopathic effect of walnut and thus alter interspecific competition in agroforestry ecosystems. Further studies are needed to analyze how juglone moves through the common mycorrhizal network from walnut to adjacent plants [30].

The results of this study also showed that AMF significantly (*P* < 0.05) triggered the increase of 2,3,5-trihydroxy-5–7-dimethoxyflavanone in walnut roots by 2.75-fold under DS condition, but not under WW condition, which was the highest fold of up-regulated metabolite. The 2,3, 5-Trihydroxy-5–7-dimethoxyflavanone is a flavonoid, and flavonoids are deemed to act as antioxidants capable of eliminating reactive oxygen species such as singlet oxygen and hydrogen peroxide in mitochondria [31]. Therefore, walnuts inoculated with AMF had a higher potential to alleviate drought-induced oxidative damage than uninoculated walnuts through flavonoids. In terms of KEGG annotation, this study also found that AMF caused oxidative phosphorylation of walnut roots under both WW and DS. Oxidative phosphorylation refers to the energy released by the oxidative step in the decomposition process of organic substances including sugars, fats, and amino acids, which leads the synthesis of ATP [32]. However, AMF spores can store ATP to tolerate abiotic stress and are induced by various stimuli [33]. Li et al. [34] also found that oxidative phosphatase was involved in *R*. *irregularis*-enhanced resistance of maize plants to low temperature, based on analysis of transcriptomics. In addition, AMF–inoculated walnut plants also represented 2.15-fold higher mannitol concentrations in roots than non-AMF plants under DS. Mannitol is an organic compound accumulated in both vacuoles and cytoplasm associated with osmotic adjustment [35]. Therefore, improved osmotic adjustment and flavonoids are involved in AMF-mediated tolerance of walnut to soil drought.

KEGG annotation in this study also showed that both phenylalanine metabolism and biosynthesis were involved in mycorrhiza-triggered metabolite responses of walnut roots under both WW and DS. Under DS, *D*. *spurca* significantly (*P* < 0.01) increased N-acetyl-L-phenylalanine concentrations in roots by 2.34-fold, and the mycorrhizal fungus triggered a significant (*P* < 0.05) increase in N-phenylacetylphenylalanine concentrations by 0.68-fold under WW, along with a 2.48-fold decrease of N-acetyl-L phenylalanine concentrations. Indeed, phenylalanine is an important precursor substance for flavonoids synthesis [36], and flavonoids are also a positive stimulant of AMF colonization and development [37]. The increase of N-Acetyl-L-phenylalanine induced by mycorrhizae under drought may accelerate the phenylalanine metabolism pathway [38], promote the formation of flavonoids, and thus maintain the establishment of mycorrhizae in the roots, which is an essential guarantee for maintaining better biomass production in mycorrhizal plants under drought. Thus, higher phenylalanine and 2,3,5-trihydroxy-5–7-dimethoxyflavanone concentrations in AMF-inoculated walnuts under drought conditions help plants remove excess reactive oxygen species, which in turn will enhance plant drought tolerance.

## Conclusion

The present study used untargeted metabolomics to reveal for the first time the secondary metabolite changes of walnut roots in response to AMF under drought conditions, and revealed the metabolic mechanism for mycorrhizal enhancement of drought tolerance in walnut. Pathway changes of differential metabolites also showed that mycorrhizae affected drought responses in a complex mechanism, and the responsive pattern was completely different from that under WW. These findings provide new insights into the enhancement of drought tolerance in walnut plants by mycorrhizae. This study also suggests that *D*. *spurca* could be appropriately introduced in walnut cultivation to enhance drought tolerance in the field, or to mycorrhizalize walnut plants at the seedling stage.

## Methods

### Arbuscular mycorrhizal fungal inoculums

Based on the results of the AMF screening conducted by Huang et al. [24] on walnuts, *Diversispora spurca* (BGC SD03A), a potentially efficient mycorrhizal fungal strain, was used in the present study. The fungal strain was supplied by the Bank of Glomales in China (BGC) (Beijing, China). This fungal strain was separated from tomato roots in Shouguang (Shandong, China) and proliferated by trap cultures using identified spores and white clover as the host plant grown in autoclaved (121 °C, 0.11 MPa, 1.5 h) substrates for 3 months. The above-ground parts of white clover were then removed from the pots, and both root segments and growth substrates were collected and used as mycorrhizal inoculums where the spore density was 26 spores/g [39]. The mycorrhizal inoculums were dried and kept at 4 °C for no more than 6 months.

### Plant culture

Seeds of *Juglans regia* L. cv. Qingxiang were provided by the Walnut Technology Promotion Center of Baokang (Hubei, China). The seeds were pre-soaked in distilled water for one week before germination, causing most of their shells to break. These seeds were then germinated at room temperature in autoclaved (121 °C, 0.11 MPa, 2 h) sand to avoid native AMF colonization. Subsequently, walnut seedlings with four leaves were transplanted into a plastic pot (2 L) with 1.85 kg of autoclaved (121 °C, 0.11 MPa, 2 h) mixture of sand and soil with a volume ratio of 1: 3. At the same time, AMF inoculation was also carried out, with 150 g of the mycorrhizal fungal inoculums per pot applied around walnut roots. The pots treated by non-AMF also received 150 g of autoclaved inoculums in addition to 2 mL of 30 µm filtrates of the fungal inoculums to keep similar microbes, except for this fungus. The inoculated seedlings were grown in a plastic greenhouse with a light intensity of 1500 Lux, air relative humidity of 65%, and 28 °C/23 °C (day/night temperature).

After 39 days of growth acclimation with 75% of maximum field water holding capacity (well-watered, WW), drought treatment began. Half of the inoculated and uninoculated plants were selected to change soil WW status to 50% of the maximum field water holding capacity (DS). Soil moisture in the other pots remained unchanged. The intensity of the drought lasted for 60 days. Soil moisture in the pots was maintained by weighing, and the lost water was supplemented at 18:00 every day to maintain the set moisture intensity. This study began in May 2021 and ended in August of the same year, for a total of 99 days. No fertilizer was applied throughout the experiment.

### Experimental design

This experiment was conducted in a completely randomized block design using inoculations with (AMF) and without (NAMF) *D*. *spurca* and two soil moisture regimes (DS and WW). There were four treatments in this experiment, and each treatment had eight replicates, for a total of 32 pots.

### Determinations of root AMF colonization rate, biomass production, and leaf water potential

At the end of the experiment, the plants were harvested, divided into aboveground and underground parts, and weighed. Then the root segments of 1-cm were cut, and mycorrhizal staining was carried out by trypan blue staining method described by Phillips and Hayman [40]. The colonization of AMF in the roots was observed under a microscope. Root AMF colonization rate was estimated by the percentage of the length of AMF-colonized root segments versus the total length of observed root segments. On a sunny day prior to harvest, the water potential was measured on the second fully expanded leaf at the top using a Dew Point Microvoltmeter (HR-33 T, Wescor Inc., Logan, Utah, USA).

### Sample extraction for metabolomic analysis

Metabolites extraction was carried out according to the protocol described by Wang et al. [41]. Simply, fresh root samples were ground to a powder under liquid nitrogen, and then 25 mg of root powder was extracted with 1 mL of the mixture of methanol, acetonitrile, and ddH_2_O (2: 2: 2, v/v) at 4 °C and 35 Hz for 1 h, accompanied by three vortexes, and centrifuged at 10,000 × *g*/min for 15 min. The supernatant obtained was dried under vacuum conditions at 37 °C, ultrasonically treated at 4 °C for 15 min, centrifuged at 13,000 × *g*/min for 15 min, and filtered through a 0.22 mm microporous membrane (Sigma) for analysis of Ultra High Performance Liquid Chromatography Q Exactive Mass Spectrometer (UHPLC-QE-MS).

### Analysis of UHPLC-QE-MS and differential metabolites

The UHPLC-QE-MS was equipped with a UPLC BEHA midecolumn (1.7 μm, 2.1 mm × 100 mm). Mobile phase A, mobile phase B and elution gradient were referred to the details described by Wang et al. [41]. Other analytical conditions were 25 °C of column temperature, 4 °C of autosampler temperature, and 2 μL of injection volume. The assay was repeated four times for each treatment.

Metabolites detected in < 20% of experimental samples or < 50% of QC samples were removed from data analysis. The total ion current of each sample was normalized. Ionic with CV > 30% in all QC samples were filtered out and excluded from downstream statistical analysis. The missing raw data were supplemented by half of the minimum value [42]. The obtained three-dimensional data involving the peak number, sample name, and normalized peak area were fed to R package metaX [43] for PCA and OPLS-DA. The metabolites with VIP (variable importance in the projection) > 1 and *P* < 0.05 (student’t test) were considered as significantly changed metabolites. The pathways of metabolites were analyzed with the databases of KEGG (http://www.kegg.jp) [44] and MetaboAnalyst (http://www.metaboanalyst.ca/).

### Statistical analysis

SAS software was used for the analysis of variance in biomass, leaf water potential and root mycorrhizal colonization rate, in which root mycorrhizal colonization rate was arcsine-transformed before analysis. Duncan's multiple range test (*P* < 0.05) was used to compare the significant differences among treatments.

## Data Availability

All data generated or analyzed during this study are included in this published article.

## Abbreviations

PCA: Principal component analysis
AMF: Arbuscular mycorrhizal fungi
WW: Well-watered
DS: Drought stress
PC: Principal component
